# The Link between Cytogenetics/Genomics and Imaging Patterns of Relapse and Progression in Patients with Relapsed/Refractory Multiple Myeloma: A Pilot Study Utilizing 18F-FDG PET/CT

**DOI:** 10.3390/cancers12092399

**Published:** 2020-08-24

**Authors:** Xiang Zhou, Alexander Dierks, Olivia Kertels, Samuel Samnick, Malte Kircher, Andreas K. Buck, Larissa Haertle, Sebastian Knorz, David Böckle, Lukas Scheller, Janin Messerschmidt, Mohammad Barakat, Marietta Truger, Claudia Haferlach, Hermann Einsele, Leo Rasche, K. Martin Kortüm, Constantin Lapa

**Affiliations:** 1 Department of Internal Medicine II, University Hospital of Würzburg, Oberdürrbacher Street 6, D-97080 Würzburg, Germany; zhou_x@ukw.de (X.Z.); haertle_l@ukw.de (L.H.); knorz_s@ukw.de (S.K.); boeckle_d@ukw.de (D.B.); scheller_l@ukw.de (L.S.); Messerschm_j@ukw.de (J.M.); barakat_m@ukw.de (M.B.); Einsele_H@ukw.de (H.E.); rasche_l@ukw.de (L.R.); Kortuem_M@ukw.de (K.M.K.); 2 Department of Nuclear Medicine, University Hospital of Würzburg, D-97080 Würzburg, Germany; dierks_a@ukw.de (A.D.); samnick_S@ukw.de (S.S.); malte.kircher@uk-augsburg.de (M.K.); buck_a@ukw.de (A.K.B.); 3 Nuclear Medicine, Medical Faculty, University Hospital of Augsburg, D-86156 Augsburg, Germany; 4 Department of Diagnostic and Interventional Radiology, University Hospital of Würzburg, D-97080 Würzburg, Germany; kertels_o@ukw.de; 5 Munich Leukemia Laboratory, D-81377Munich, Germany; marietta.truger@mll.com (M.T.); claudia.haferlach@mll.com (C.H.)

**Keywords:** radiogenomics, 18F-FDG PET/CT, multiple myeloma, relapse, progression, pattern

## Abstract

Utilizing 18F-fluorodeoxyglucose (18F-FDG) positron emission tomography (PET)/computed tomography (CT), we performed this pilot study to evaluate the link between cytogenetic/genomic markers and imaging patterns in relapsed/refractory (RR) multiple myeloma (MM). We retrospectively analyzed data of 24 patients with RRMM who were treated at our institution between November 2018 and February 2020. At the last relapse/progression, patients had been treated with a median of three (range 1–10) lines of therapy. Six (25%) patients showed FDG avid extramedullary disease without adjacency to bone. We observed significantly higher maximum standardized uptake values (SUV_max_) in patients harboring del(17p) compared with those without del(17p) (*p* = 0.025). Moreover, a high SUV_max_ of >15 indicated significantly shortened progression-free survival (PFS) (*p* = 0.01) and overall survival (OS) (*p* = 0.0002). One female patient exhibited biallelic *TP53* alteration, i.e., deletion and mutation, in whom an extremely high SUV_max_ of 37.88 was observed. In summary, this pilot study suggested a link between del(17p)/*TP53* alteration and high SUV_max_ on 18F-FDG PET/CT in RRMM patients. Further investigations are highly warranted at this point.

## 1. Introduction

**Figure 1:**
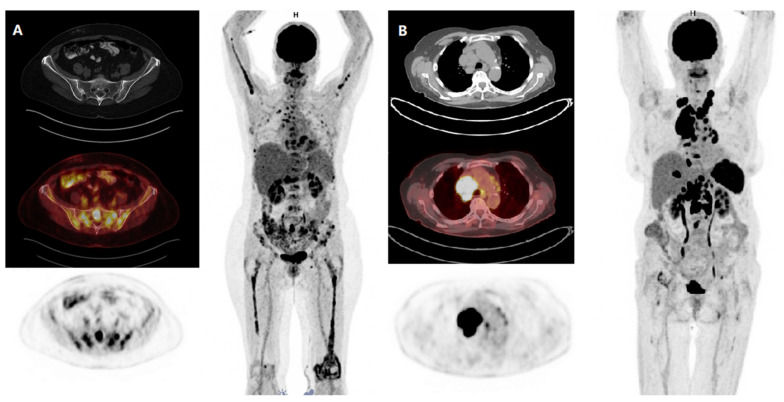
18F-FDG PET/CT in two patients with multiple myeloma. 18F-FDG PET/CT demonstrates FDG avid (**A**) medullary lesions (pelvis, spine, both humeri and both femurs) and (**B**) extramedullary manifestations (mediastinal lymph nodes) in patients with multiple myeloma. 18F-FDG, 18F-fluorodeoxyglucose; PET, positron emission tomography; CT, computed tomography.

Multiple myeloma (MM) represents the second most common hematological malignancy in adults [1]. In MM, functional imaging methods, such as diffusion-weighted (DW) magnetic resonance imaging (MRI) and 18F-fluorodeoxyglucose (18F-FDG) positron emission tomography (PET)/computed tomography (CT), can display diffuse growth patterns, focal lesions, and extramedullary disease (EMD) of patients (Figure 1). In the current consensus recommendation by the International Myeloma Working Group (IMWG), 18F-FDG PET/CT is considered a valuable tool for the visualization of disease activity in both newly diagnosed (ND) and relapsed/refractory (RR) MM patients [2]. 18F-FDG PET/CT has also been used for the prediction of survival outcome in MM patients treated with allogeneic stem cell transplant [3].

To date, there are also a few studies evaluating the association between cytogenetic abnormalities and imaging patterns in newly diagnosed MM (NDMM). Recently, adverse cytogenetics, such as del(17p), gain(1q21), and t(4;14), have been reported to be enriched in NDMM patients with diffuse infiltration pattern in DWMRI C [4]. Moreover, del(17p), gain(1q21), and gene expression profiling (GEP)-based high-risk disease are more frequent in NDMM patients with at least three large focal lesions >5 cm^2^ [5]. Furthermore, McDonald et al. reported that total lesion glycolysis (TLG) >620 g and metabolic tumor volume (MTV) >210 cm^3^ indicated a significantly inferior progression-free survival (PFS) and overall survival (OS) of myeloma patients [6]. However, it has been less extensively investigated if imaging patterns of relapse and progression correlate with cytogenetic/genomic markers in RRMM. Therefore, we performed this pilot study in RRMM utilizing 18F-FDG PET/CT.

The aim of the current study was to evaluate the potential link between imaging patterns of relapse or progression and cytogenetic/genomic characteristics in RRMM and to generate hypotheses for further investigations.

## 2. Methods

### 2.1. Patient Population

This was a single-center retrospective cohort study. We identified 24 patients who were treated for RRMM between November 2018 and February 2020. RRMM was defined as per current IMWG criteria [7]. At the last relapse/progression, we simultaneously performed a bone marrow biopsy plus an 18F-FDG-PET/CT prior to therapy initiation in all patients. Patients with active second tumor were excluded from the analysis. Patients’ characteristics, including time point of diagnosis, MM subtype, prior lines of therapy, and drug resistance status, were collected for the analysis of imaging data (PET/CT scans and DWMRI if available). In addition, patterns of relapse or disease progression were noted (presence of extramedullary disease, serological activity, bone marrow infiltration rate, cytogenetics, and genomic data). All procedures were performed in accordance with national ethical standards and with the current version of the Declaration of Helsinki.

### 2.2. Imaging Analysis, Cytogenetics, and Genomic Data

We assessed the numbers of medullary and extramedullary sites, maximum standardized uptake value (SUV_max_) of lesions, and the localization of the largest and “hottest” lesion. Correlation with DWMRI was performed in patients with available imaging. More details of 18F-FDG PET/CT image acquisition and imaging analysis are available in the Supplementary Methods.

Cytogenetic and genomic analyses were performed using bone marrow materials collected at the last relapse or progression. Cytogenetics was analyzed by fluorescence in situ hybridization (FISH) on CD138 purified cells. High-risk cytogenetics was defined according to the revised international staging system (R-ISS) for MM, i.e., del(17p), t(4;14), and t(14;16) [8]. Structural variations (SV), copy number variations (CNV), and point mutations were available from whole-genome sequencing (WGS) on CD138 purified cells in nine cases. More details are available in the 8F-FDG PET/CT image acquisition and imaging analysis are available in the Supplementary Methods or upon reasonable request.

### 2.3. Statistical Analysis

We summarized patients’ characteristics as absolute number and percentage or as median and range if not otherwise stated. Two-tailed Mann–Whitney U test was used to compare the SUV values in different subgroups. We used Kaplan–Meier methods to analyze the survival outcome of the patients. A univariate log-rank test was used to compare the survival curves in different groups. These analyses were performed with GraphPad Prism 5.0. A *p*-value of <0.05 was considered statistically significant.

## 3. Results

### 3.1. Patients’ Characteristics

All 24 patients suffered from relapse or progression of MM at the time point of bone marrow biopsy and 18F-FDG-PET/CT, which were performed prior to therapy initiation. Overall, 58% of the patients (*n* = 14) were male, and the median age at the last relapse/progression was 68 (range 46–81) years. The median time between diagnosis of MM and the last relapse/progression was 62 (range 17–192) months. Our cohort was highly pretreated with a median of three (range 1–10) prior lines of therapy. Most patients (*n* = 23, 96%) underwent high-dose melphalan and autologous, and three (13%) patients also allogeneic stem cell transplant (SCT). All patients (100%) had received prior bortezomib, and ten of them (42%) additional carfilzomib treatment. Lenalidomide, pomalidomide, and thalidomide were administered in 20 (83%), seven (29%), and three (13%) patients, respectively. Daratumumab was given in 13 (54%) patients, and two (8%) patients received elotuzumab. Eight (33%), nine (38%), 14 (58%), six (25%), and 11 (46%) patients were bortezomib, carfilzomib, lenalidomide, pomalidomide, and daratumumab refractory, respectively, and three (13%) patients were penta-refractory (bortezomib, carfilzomib, lenalidomide, pomalidomide, and daratumumab). Moreover, one (4%) and two (8%) patients received B-cell maturation antigen (BCMA)-targeted chimeric antigen receptor (CAR) T-cell therapy and bispecific antibody within clinical trials, respectively.

At the initial diagnosis of MM, all patients (100%) had measurable M component in serum, and primary EMD was present in five (21%) patients. In addition, one (4%) patient suffered from primary plasma cell leukemia (PCL) with 19.3 × 10^3^/µL circulating plasma cells in peripheral blood at diagnosis. Patients’ characteristics and treatment-related data are summarized in Table 1.

### 3.2. Patterns of Relapse and Progression

Fourteen (58%) patients progressed on or within 60 days of receiving the last treatment, and we then started a new line of therapy. The other ten (42%) patients relapsed from partial remission (PR) or better, and the median time after the last treatment was 10 (range 3–29) months in these ten patients. The majority of the patients (*n* = 22, 92%) presented an increasing M component in serum, while two (8%) of them showed no serological activity but EMD. Four (17%) patients had a bone marrow infiltration of <10%. The lactate dehydrogenase level was elevated in seven (29%) patients. Thirteen (54%) patients showed a β2-microglobulin level of ≥3.5 mg/L.

As demonstrated in 18F-FDG PET/CT, which was performed at the last relapse/progression, the vast majority of the patients (*n* = 23, 96%) exhibited medullary lesions. Six (25%) patients suffered from true EMD without adjacency to bone. The lymph node was the most common localization of EMD (3/6). One and two out of six patients had EMD in soft tissue and parenchymal organ, respectively. At the time point of the last relapse/progression, the one patient who had primary PCL at the first diagnosis developed soft tissue EMD and serological progression. However, PCL was no longer present in this patient. Among all medullary and extramedullary lesions, the median SUV_max_ was 8.15 (range 3.81–39.14). In two (8%) patients, EMD represented the overall hottest lesion. At the last relapse/progression, both 18F-FDG PET/CT scan and DWMRI were available in six (25%) patients. Notably, in two out of six patients, we observed more diffusion-weighted imaging (DWI) positive lesions in DWMRI compared to 18F-FDG PET/CT scans. Patterns of relapse and progression are summarized in Table 2.

### 3.3. Cytogenetics and Imaging Patterns of Relapse and Progression

We analyzed the link between cytogenetics and imaging patterns of relapse and progression, which were demonstrated in 18F-FDG PET/CT scans. Cytogenetics obtained at the last relapse/progression was available in 23 patients. High-risk cytogenetics, as determined by FISH, was present in eight (33%) patients, with four (17%), five (21%), one (4%) patients harboring del(17p), t(4;14), and t(14;16), respectively. Ten (42%) patients exhibited gain(1q21). Fifteen (63%) patients had standard-risk cytogenetics. EMD prevalence in patients with high-risk cytogenetics was slightly higher than that with standard-risk cytogenetics (2/8, 25% versus 3/15, 20%). In the patient with primary PCL and soft tissue EMD progression, we did not find any unfavorable cytogenetic alterations, such as t(4;14), del(17p), or gain(1q21), and the patient exhibited t(11;14). Among the three penta-refractory patients, two of them had hyperdiploid myeloma, and one patient displayed t(11;14) and gain(1q21).

**Figure 2:**
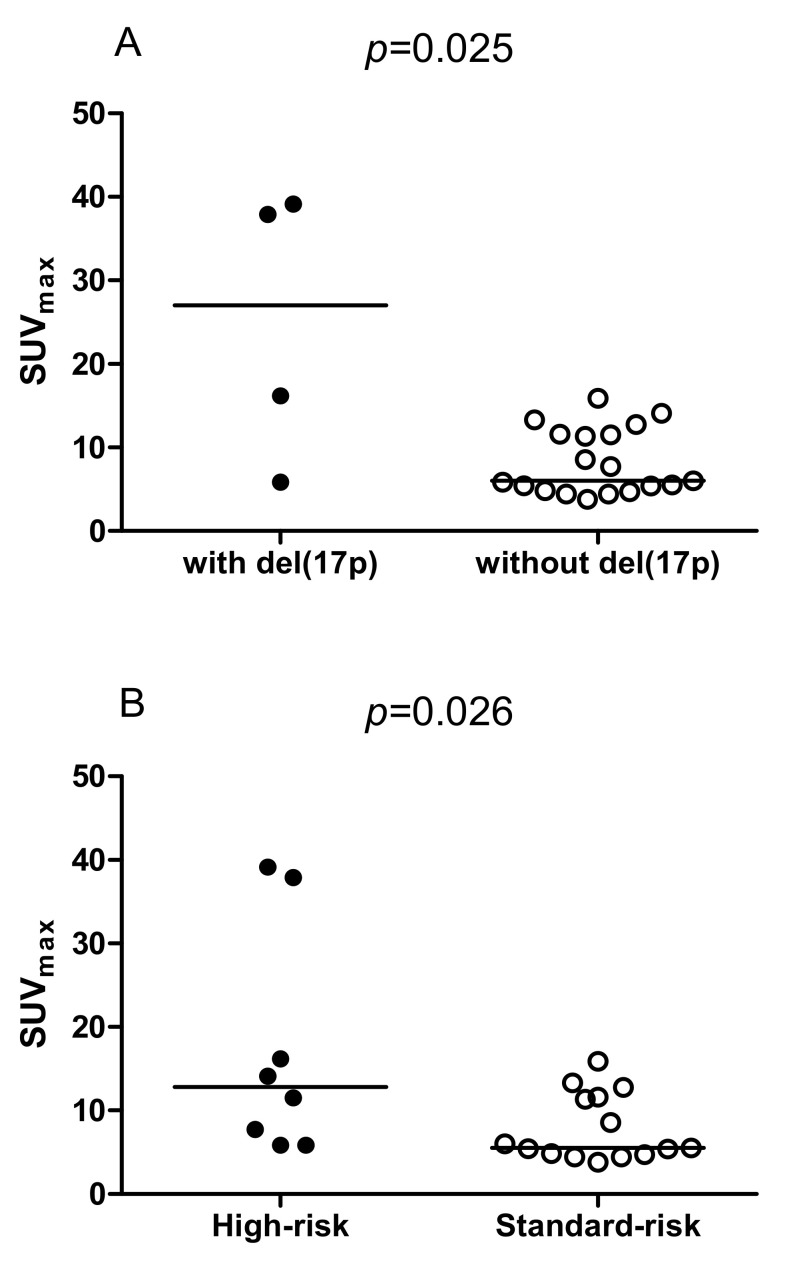
Link between cytogenetics and maximum standardized uptake value (SUV_max_): (**A**) Patients with del(17p) (*n* = 4) showed a significantly higher SUV_max_ compared with those without del(17p) (*n* = 19) (*p* = 0.025). (**B**) High-risk cytogenetics, i.e., t(4;14), t(14;16), and del(17p) (*n* = 8) indicated a significantly higher SUV_max_ when compared with standard-risk cytogenetics (*n* = 15) (*p* = 0.026).

**Figure 3:**
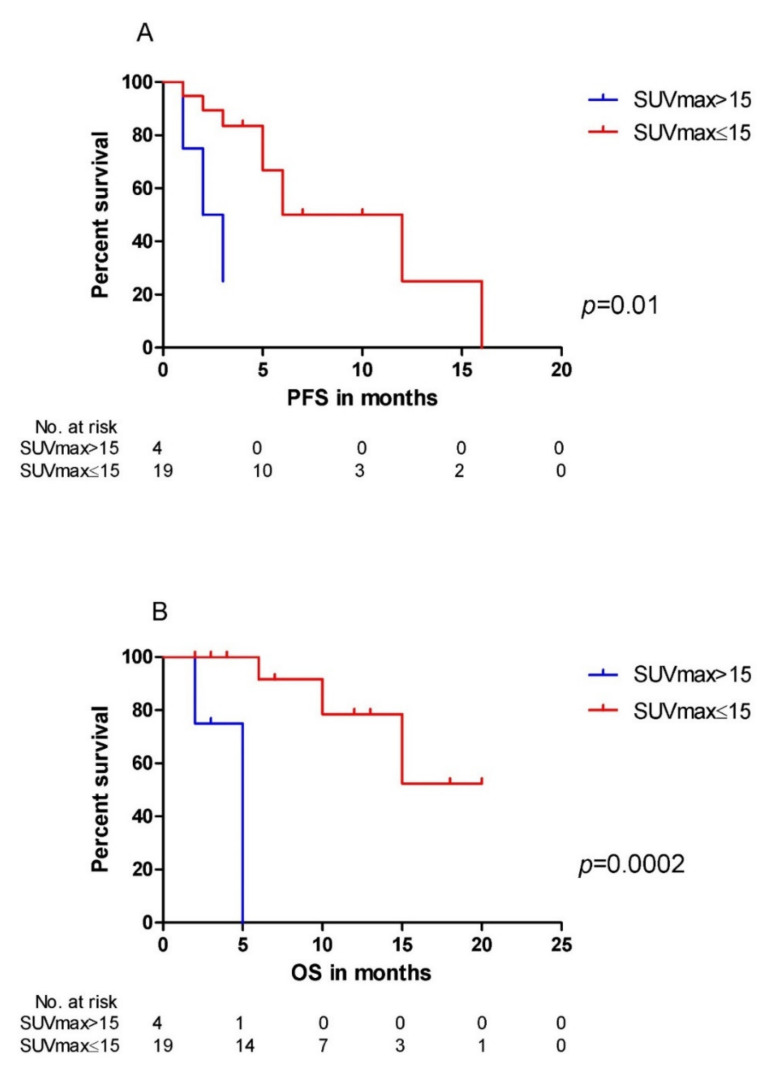
Maximum standardized uptake value (SUV_max_) and survival: (**A**) Progression free survival (PFS) of patients with SUV_max_ >15 (*n* = 4) was significantly shorter than that in patients with SUV_max_ ≤15 (*n* = 19) (*p* = 0.01). (**B**) Patients with SUV_max_ >15 (*n* = 4) had a significantly inferior overall survival (OS) compared to those with SUV_max_ ≤15 (*n* = 19) (*p* = 0.0002).

We observed a significantly higher SUV_max_ in patients harboring del(17p) when compared with those without del(17p) (median SUV_max_: 27.03 versus 6.04, *p* = 0.025, Figure 2A). Moreover, patients with high-risk cytogenetics showed a significantly higher SUV_max_ in comparison with those with standard-risk cytogenetics (median SUV_max_: 12.80 versus 5.54, *p* = 0.026, Figure 2B). Furthermore, we observed no difference in SUV_max_ between patients with and without gain(1q21) (*p* = 0.200, figure not shown). Importantly, a high SUV_max_ of >15 indicated a significantly inferior PFS (*p* = 0.01, Figure 3A) and OS (*p* = 0.0002, Figure 3B) in our patients with RRMM.

### 3.4. WGS and Imaging Patterns of Relapse and Progression

To further elucidate the relationship between genomic alterations and imaging patterns, we also reviewed the data of WGS at the last relapse/progression, which were available in nine out of 24 patients. In these nine patients, WGS data could confirm structural changes within the genome, including translocations, amplifications, and deletions, which had been detected by FISH analysis at the last relapse/progression. Overall, t(14;16) and gain(1q21) were present in one (11%) and five (56%) patients, respectively. One (11%) patient exhibited del(17p). With regard to gene mutation status, *KRAS* represented the most frequently mutated gene in our cohort (*n* = 4, 44%), followed by *NRAS* (*n* = 2, 22%) mutation. WGS data and the patients’ characteristics are presented in Table 3.

**Figure 4:**
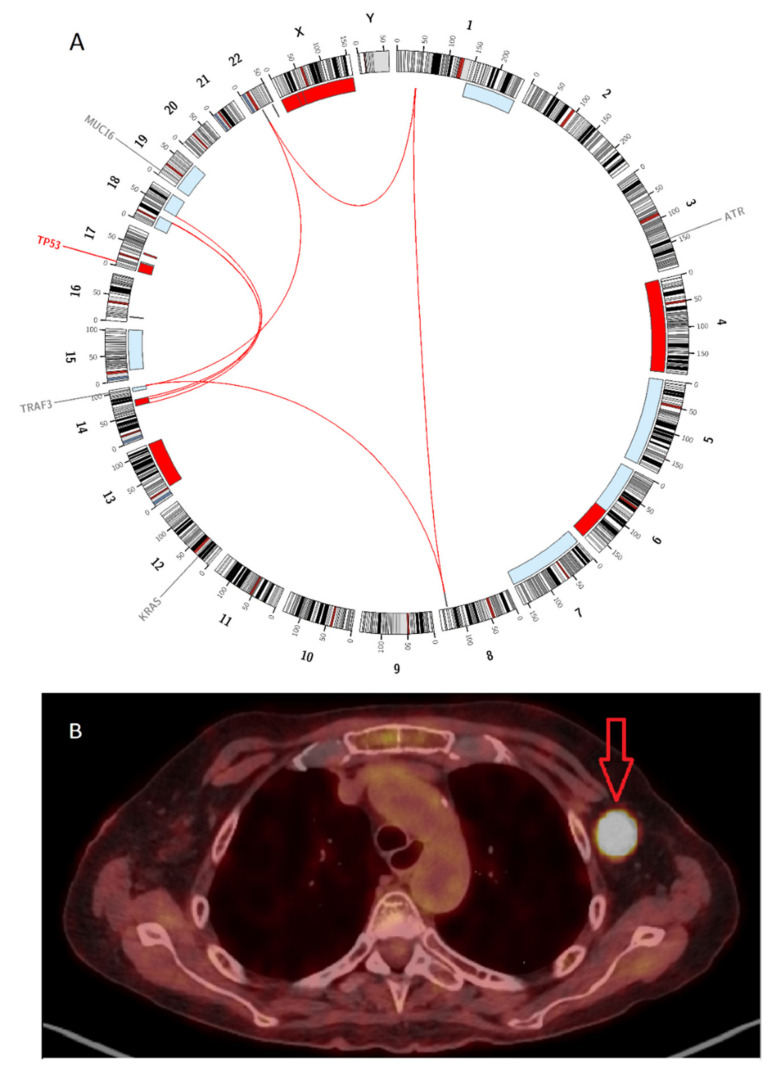
Whole-genome sequencing (WGS) and 18F-FDG PET/CT in the patient with biallelic *TP53* alteration (No. 5 in Table 3): (**A**) Circos plot demonstrated data of WGS, including copy number variations (CNV), structural variations (SV), and single nucleotide variations (SNV), at the last relapse. Gains and losses of >1 Mb are shown in blue and red, respectively. Interchromosomal reciprocal translocations with variant allele frequency (vaf) >0.1 are displayed by red lines inside the circle. Gene mutations (*TP53*) and variants (*ATR*, *KRAS*, *TRAF3,* and *MUC16*) are marked in red and grey, respectively. (**B**) True extramedullary disease (axillary lymph node) with excessive FDG uptake, as shown on 18F-FDG PET/CT scans. Biallelic *TP53* alteration might lead to aggressive tumor growth, e.g., development of extramedullary disease (EMD) and excessive FDG uptake.

Notably, there was one female patient with biallelic *TP53* alteration (patient No. 5 in Table 3). In this patient, a del(17p) was found by FISH analysis. The WGS data revealed a *TP53* mutation ENST00000269305.4:c.375 + 1G > T with a variant allele frequency (vaf) of 84% in one allele and a loss of the remaining allele through deletion chr17_p13.3_1::18986000_p11.2 (Figure 4A). At the last relapse/progression, this patient suffered from serological disease progression with EMD in lymph nodes. She received an allogeneic SCT as salvage therapy, and, two months later, this patient developed a new true EMD lesion in lymph node with excessive FDG uptake (Figure 4B). Interestingly, in 18F-FDG PET/CT scans, this patient also showed the highest SUV_max_, which was much higher than the other eight patients (Table 3). This finding was consistent with our results demonstrated by FISH analysis that del(17p) indicated a significantly higher SUV_max_ at relapse/progression in 18F-FDG PET/CT scans in RRMM patients compared with those without del(17p).

## 4. Discussion

We performed this pilot study utilizing 18F-FDG PET/CT to explore the potential link between cytogenetic/genomic characteristics and imaging patterns of relapse and progression in MM. To the best of our knowledge, this is the first study analyzing this link in patients with RRMM.

Overall, in our cohort, a high SUV_max_ of >15 on 18F-FDG PET/CT scans indicated significantly inferior PFS and OS in patients with relapsed or progressive MM. In 18F-FDG PET/CT, generally, SUV_max_ is a semi-quantitative parameter correlated with glucose uptake and metabolic or proliferative activity of the tumor [9]. So far, published data on the prognostic role of SUV_max_ in RRMM are still very limited [2]. Recently, in another study of Jamet *et al*., SUV_max_ of >15.9 was identified as an independent negative prognostic factor for PFS [10] in patients with relapsed MM. In addition, Lapa et al. found that SUV_max_ of >18.57 was predictive for a shorter time to progression (TTP) in patients with MM relapse after autologous SCT [11]. In our study, we took a comparable cut-off value of SUV_max_ (>15), as already reported, and our results were in line with these previous studies. These findings underline the prognostic value of SUV_max_ on 18F-FDG PET/CT scans for RRMM patients.

As yet, little is known about the link between cytogenetics and semi-quantitative parameters in 18F-FDG PET/CT scans, such as SUV_max_ in RRMM patients. In our cohort, RRMM patients with high-risk cytogenetics, including del(17p), showed a significantly higher SUV_max_ in 18F-FDG PET/CT scans compared with those with standard-risk cytogenetics. More importantly, among the four patients with SUV_max_ of >15, three of them (3/4) showed del(17p) and, consequently, also high-risk cytogenetics. As reported by IMWG in the R-ISS in 2015, primary genetic events t(4;14), t(14;16) and secondary genetic abnormality del(17p) are known as negative prognostic factors in MM, and R-ISS is one of the most widely used prognostic models worldwide [8,12]. Additionally, in a study of Zamagni et al., (1) high-risk cytogenetics, i.e., del(17p) and t(4;14), and (2) the presence of lesions with SUV > 4.2 were identified as negative prognostic factors for PFS in NDMM [13]. At this point, our study demonstrated the prognostic values of high-risk cytogenetics and high SUV_max_ in RRMM and elucidated the link between both prognostic factors, suggesting that imaging parameters, such as SUV_max_, might be a potential surrogate marker of cytogenetics in RRMM. Similar to previous studies, EMD was also enriched in patients with high-risk cytogenetics in our cohort [14,15]. However, these findings should be interpreted with caution due to the small patient population in our analysis. Altogether, the current study demonstrated that both SUV_max_ and cytogenetics, probably due to the potential link between both factors, were predictive for the survival outcome of RRMM patients.

In our cohort, WGS data revealed a patient with biallelic *TP53* alteration, in whom an extremely high SUV_max_ of 37.88 was presented by 18F-FDG PET/CT scans. *TP53* is a well-known tumor suppressor gene, and its dysfunction is associated with various malignant diseases in humans [16]. In this patient, we detected a *TP53* mutation ENST00000269305.4:c.375 + 1G > T, which had been reported in ovarian cancer and breast cancer in the International Agency for Research on Cancer (IARC) *TP53* Mutation Database [17]. Currently, the role of this SNV is not fully understood. Mutations in this region could affect a splice site in intron 4 of *TP53* and might result in a frameshift and probably the loss of *TP53* function [18]. Thus, this SNV has been classified as a pathogenic variant in the Catalogue of Somatic Mutations in Cancers (COSMIC) database (Legacy Identifier: COSM69405). In addition, we observed a loss of the remaining *TP53* allele due to a large deletion chr17_p13.3_1::18986000_p11.2. Taken together, this patient presented a so-called double-hit *TP53* alteration, i.e., mutation plus deletion, which might result in a severe deficiency of *TP53* function. Interestingly, we observed the highest FDG uptake in an EMD lesion (SUV_max_ 37.88), indicating an extremely high metabolic and proliferative activity of EMD. Biallelic *TP53* alteration might correlate with aggressive behaviors of MM, e.g., development of EMD and excessive FDG uptake. This finding should be further evaluated in larger studies.

The current pilot study had several limitations: (1) In our study, we selected 18F-FDG PET/CT scans as a candidate parameter to elucidate the link between cytogenetics/genomics and imaging patterns in RRMM. While 18F-FDG clearly is the standard of reference tracer in nuclear imaging of MM, scan results might be influenced by different factors, such as expression levels of hexokinase-2 and glucose transporter, as well as hyperglycemia, and false-positive results due to infection, chronic inflammation, metallic implants, surgery, and fracture healing can occur [2,19,20,21,22]. In this context, the hexokinase-2 expression is increased in the HY and PR molecular subgroup [19]. In principle, additional semi-quantitative parameters, such as MTV and TLG, or PET/CT using other tracers, such as 11C-methionine [23,24] and 68Ga-Pentixafor [25,26], can also be used. A combination of different imaging methods might help to reduce the opportunities for bias. (2) Additional gene analysis of EMD lesions, if available, should also be performed to further evaluate the link between imaging patterns and special cytogenetic/genomic features of EMD [27]. (3) Our patients had received heterogeneous pretreatment, which might impact the clonal evolution and, consequently, also the genetic/genomic profile of MM cells. (4) As our pilot study was a retrospective study based on a limited number of patients, we did not perform multivariate survival analysis, and our findings should be interpreted with caution. Nevertheless, our findings have given insight into the biological background of imaging patterns in RRMM and have provided a rationale for further investigations.

## 5. Conclusions

In conclusion, this pilot study suggested a link between del(17p)/*TP53* alteration and FDG-uptake on FDG PET/CT scans in RRMM patients. Further larger studies are highly warranted at this point.

## Figures and Tables

**Table 1:** Patients’ characteristics.

Parameter	Number
**Patients, *n***	24
**Gender, *n* (%)**	
Male	14 (58)
Female	10 (42)
**Age at the last relapse/progression, median (range), years**	68 (46–81)
**Subtype, *n* (%)**	
IgG	16 (67)
IgA	7 (29)
LC	1 (4)
**ISS Stage, *n* (%)**	
I	9 (37)
II	5 (21)
III	5 (21)
NA	5 (21)
**Cytogenetics, *n* (%)**	
High-risk	8 (33)
Standard-risk	15 (63)
NA	1 (4)
**t(4;14)**	
Yes	5 (21)
No	18 (75)
NA	1 (4)
**t(14;16)**	
Yes	1 (4)
No	19 (79)
NA	4 (17)
**del(17p)**	
Yes	4 (17)
No	19 (79)
NA	1 (4)
**gain(1q21)**	
Yes	10 (42)
No	13 (54)
NA	1 (4)
**EMD at diagnosis, *n* (%)**	
Yes	5 (21)
No	19 (79)
**Prior lines of therapy, *n* (%)**	
2–1 month	9 (38)
4–3 month	7 (29)
≥5	8 (33)
**Prior treatments, *n* (%)**	
**PIs**	
Bortezomib	24 (100)
Carfilzomib	10 (42)
**IMiDs**	
Lenalidomide	20 (83)
Pomalidomide	7 (29)
Thalidomide	3 (13)
**Monoclonal antibodies**	
Daratumumab	13 (54)
Elotuzumab	2 (8)
**SCT**	
Prior autologous SCT	23 (96)
Prior allogenic SCT	3 (13)
**BCMA-directed novel immunotherapies within clinical trials**	
Bispecific antibody	2 (8)
CAR-T-cell	1 (4)
**Drug resistance, *n* (%)**	
Bortezomib	8 (33)
Carfilzomib	9 (38)
Lenalidomide	14 (58)
Pomalidomide	6 (25)
Daratumumab	11 (46)

BCMA—B-cell maturation antigen; CAR—chimeric antigen receptor; EMD—extramedullary disease; IMiDs—immunomodulatory drugs; ISS—the multiple myeloma international staging system; LC—light chain; MM—multiple myeloma; NA—not available; PIs—proteasome inhibitors; SCT—stem cell transplant.

**Table 2:** Patterns of relapse and progression.

Parameter	Number
**Patients, *n***	24
**Serological activity, *n* (%)**	
Yes	22 (92)
No	2 (8)
**Bone marrow infiltration, *n* (%)**	
<10%	4 (17)
≥10%	17 (71)
NA	3 (12)
**Laboratory parameters, *n* (%)**	
**eGFR, mL/min (CKD-EPI), median (range)**	70 (34–98)
≥50 mL/min	18 (75)
<50 mL/min	6 (25)
**Calcium, mmol/L, median (range)**	2.5 (2.0–2.3)
≥2.5 mmol/L	0 (0)
<2.5 mmol/L	24 (100)
**LDH, U/L, median (range)**	197 (107–711)
≥250 U/L	7 (29)
<250 U/L	17 (71)
**Hemoglobin, g/dL, median (range)**	11.0 (7.7–14.3)
≥10 g/dL	16 (67)
<10 g/dL	8 (33)
**β2-microglobulin, mg/L, median (range)**	3.6 (1.7–9.7)
≥3.5 mg/L	13 (54)
<3.5 mg/L	11 (46)
**Number of medullary lesions, *n* (%)**	
0	1 (4)
3–1 month	6 (25)
7–4 month	2 (8)
>7	15 (63)
**Number of EMD, n (%)**	
0	18 (75)
3–1 month	4 (17)
7–4 month	1 (4)
>7	1 (4)
**Localization of EMD, *n* (%)**	
Lymph node	3 (12)
Parenchymal organ	2 (8)
Soft tissue	1 (4)
**SUV_max_, median (range)**	8.15 (3.81–39.14)
**Localization of the hottest lesion, *n* (%)**	
Medullary	22 (92)
Extramedullary	2 (8)
**Comparison between DWMRI and 18F-FDG-PET/CT (*n* = 6), *n* (%)**	
Number of DWI positive lesions > FDG avid lesions	2 (33)
Number of DWI positive lesions < FDG avid lesions	1 (17)
Number of DWI positive lesions = FDG avid lesions	3 (50)

18F-FDG-PET/CT—18F-fluorodeoxyglucose positron emission tomography/computed tomography; CKD-EPI—Chronic Kidney Disease Epidemiology Collaboration; eGFR—estimated glomerular filtration rate; DWI—diffusion-weighted imaging; DWMRI—diffusion-weighted magnetic resonance imaging; EMD—extramedullary disease; LDH—lactate dehydrogenase; NA—not available; SUV_max_—maximum standardized uptake value.

**Table 3:** Whole-genome sequencing and imaging patterns in 18F-FDG PET/CT scans.

Patient	Gender	Age at Diagnosis	Subtype	Lines of Prior Therapy	Mutated Genes	High-Risk Structural Alterations	SUV_max_	EMD	Size of the Largest EMD, cm	Localization of the Largest EMD
1	M	54	IgA Kappa	5	*KRAS*	None	5.4	No	/	/
2	F	75	IgG Kappa	3	*KRAS*, *CUL4B*	None	12.78	Yes	2.6	Lymph node
3	M	48	IgG Kappa	3	*BRAF*	None	4.44	No	/	/
4	M	78	IgG Kappa	1	None	None	8.58	No	/	/
5	F	60	IgG Kappa	7	*TP53*	del(17p), gain(1q21)	37.88	Yes	3.1	Lymph node
6	M	66	IgA Kappa	2	*KRAS*, *MUC16*, *NRAS*, *RRBP1*, *FAM46C*	gain(1q21)	11.36	No	/	/
7	F	64	IgA Kappa	8	*KRAS*	gain(1q21)	6.04	No	/	/
8	F	74	IgA Lambda	2	None	t(14;16), gain(1q21)	7.73	No	/	/
9	M	72	Kappa LC	1	*NRAS*	gain(1q21)	13.31	No	/	/

18F-FDG-PET/CT—18F-fluorodeoxyglucose positron emission tomography/computed tomography; EMD—extramedullary disease; F—female; LC—light chain; M—male; SUV_max_—maximum standardized uptake value.

## Supplementary Materials

The following are available online at https://www.mdpi.com/2072-6694/12/9/2399/s1, Supplementary Methods.
