# Supplementary material for: The Link between Cytogenetics/Genomics and Imaging Patterns of Relapse and Progression in Patients with Relapsed/Refractory Multiple Myeloma: A Pilot Study Utilizing 18F-FDG PET/CT
Source: Cancers (Basel). 2020 Aug 24;12(9):2399. doi: 10.3390/cancers12092399 (PMC7564496; doi:10.3390/cancers12092399)
Supplement: Supplementary file 1 [file cancers-12-02399-s001.pdf]

cancers MDPI

Supplementary methods

1. Tracer Synthesis and PET

Imaging for all patients was performed on an integrated PET/CT scanner (Siemens Biograph mCT 64, Siemens Healthineers, Knoxville, USA). Prior to imaging, patients fasted for at least 6 h. Blood glucose levels were measured and confirmed to be below 130 mg/dl before the intravenous injection of 150 - 300 MBq FDG. After a distribution period of 60 min, PET emission data were acquired in 3D-mode with a 200 × 200 matrix with 2 min emission time per bed position from the vertex of the skull to the knees. Consecutively, transmission data were acquired using a low-dose protocol (CARE Dose 4D; 80 mAs; 120 kV; matrix: 512 × 512; 3 mm slice thickness; increment: 30 mm/s; rotation time: 0.5 s; pitch index: 0.8) or a full-dose protocol (dose modulation with a quality reference of 180 mAs, 120 kV, a 512 × 512 matrix, 5-mm slice thickness, increment of 30 mm/s, rotation time of 0.5 s, and pitch index of 1.4). PET data were reconstructed iteratively (3 iterations, 24 subsets, Gaussian filtering of 2.0 mm full width at half maximum) with attenuation correction using dedicated standard with attenuation correction using dedicated standard software (HD. PET, Siemens Esoft, Siemens Healthineers, Erlangen, Germany).

2. Analysis of Imaging

First, a visual inspection of scans for FDG uptake in medullary and extramedullary lesions was performed. Then, a spherical volume of interest was drawn to include respectively the biggest medullary and extramedullary lesion as well as the lesion with the maximum tracer uptake. Standardized uptake values for maximal (SUV_{max}) and mean tumor uptake (SUV_{mean}) were automatically calculated by a dedicated software package (syngo.via workstation, Siemens Healthineers).

3. Whole Genome Sequencing

WGS was performed on CD138 purified cells. Library preparation was performed using TruSeq DNA PCR-Free HT sample preparation kit (Illumina, San Diego, CA, USA) according to manufacturer's protocol and 151 bp paired-end sequences were generated on NovaSeq6000 sequencing instruments (Illumina) with 100x coverage. As matched-normal samples were not available, a mixture of genomic DNA from multiple anonymous donors was used as normal controls. Read mapping and tumor/normal variant calling were performed using Illumina's WGS app version 5.0 and tumor normal app 3.0 for analysis of genomic data. WGS reads were mapped to human reference genome (Ensembl GRCh37) using Ilumina's Isaac aligner (iSAAC-03.16.02.19). Structural variations (SV) were called using Manta (version 0.28.0) and only passed variants with >2 paired reads were considered for further analysis. Copy number variations (CNV) calling was performed using GATK4 following Broad's best practices recommendation. For illustration of SV and CNV via circos plot only interchromosomal reciprocal fusions with vaf >0.1 and CNV >1 Mb were included. Strelka2 variant caller was used for single nucleotide variations (SNV). To remove potential germline variants, each variant was queried against the gnomAD database, variants with global population frequencies >1% were excluded.
